# Transcription signatures encoded by ultraconserved genomic regions in human prostate cancer

**DOI:** 10.1186/1476-4598-12-13

**Published:** 2013-02-14

**Authors:** Robert S Hudson, Ming Yi, Natalia Volfovsky, Robyn L Prueitt, Dominic Esposito, Stefano Volinia, Chang-Gong Liu, Aaron J Schetter, Katrien Van Roosbroeck, Robert M Stephens, George A Calin, Carlo M Croce, Stefan Ambs

**Affiliations:** 1 Laboratory of Human Carcinogenesis, Center for Cancer Research (CCR), National Cancer Institute (NCI), National Institutes of Health, Bethesda, MD, USA; 2 Advanced Biomedical Computing Center, SAIC-Frederick, Inc., NCI, Frederick, MD, USA; 3 Protein Expression Laboratory, Advanced Technology Program, SAIC-Frederick, Inc., NCI, Frederick, MD, USA; 4 Department of Molecular Virology, Immunology and Medical Genetics, Comprehensive Cancer Center, Ohio State University, Columbus, OH, USA; 5 Department of Experimental Therapeutics, The University of Texas MD Anderson Cancer Center, Houston, TX, USA

**Keywords:** Ultraconserved region, Gene expression, Prostate cancer

## Abstract

**Background:**

Ultraconserved regions (UCR) are genomic segments of more than 200 base pairs that are evolutionarily conserved among mammalian species. They are thought to have functions as transcriptional enhancers and regulators of alternative splicing. Recently, it was shown that numerous RNAs are transcribed from these regions. These UCR-encoded transcripts (ucRNAs) were found to be expressed in a tissue- and disease-specific manner and may interfere with the function of other RNAs through RNA: RNA interactions. We hypothesized that ucRNAs have unidentified roles in the pathogenesis of human prostate cancer. In a pilot study, we examined ucRNA expression profiles in human prostate tumors.

**Methods:**

Using a custom microarray with 962 probesets representing sense and antisense sequences for the 481 human UCRs, we examined ucRNA expression in resected, fresh-frozen human prostate tissues (57 tumors, 7 non-cancerous prostate tissues) and in cultured prostate cancer cells treated with either epigenetic drugs (the hypomethylating agent, 5-Aza 2^′^deoxycytidine, and the histone deacetylase inhibitor, trichostatin A) or a synthetic androgen, R1881. Expression of selected ucRNAs was also assessed by qRT-PCR and NanoString®-based assays. Because ucRNAs may function as RNAs that target protein-coding genes through direct and inhibitory RNA: RNA interactions, computational analyses were applied to identify candidate ucRNA:mRNA binding pairs.

**Results:**

We observed altered ucRNA expression in prostate cancer (e.g., uc.106+, uc.477+, uc.363 + A, uc.454 + A) and found that these ucRNAs were associated with cancer development, Gleason score, and extraprostatic extension after controlling for false discovery (false discovery rate < 5% for many of the transcripts). We also identified several ucRNAs that were responsive to treatment with either epigenetic drugs or androgen (R1881). For example, experiments with LNCaP human prostate cancer cells showed that uc.287+ is induced by R1881 (*P* < 0.05) whereas uc.283 + A was up-regulated following treatment with combined 5-Aza 2^′^deoxycytidine and trichostatin A (*P* < 0.05). Additional computational analyses predicted RNA loop-loop interactions of 302 different sense and antisense ucRNAs with 1058 different mRNAs, inferring possible functions of ucRNAs via direct interactions with mRNAs.

**Conclusions:**

This first study of ucRNA expression in human prostate cancer indicates an altered transcript expression in the disease.

## Background

An ultraconserved region (UCR) is a genomic sequence with 100% conservation between human, rat, and mouse genomes. At more than 200 base pairs in length, 481 of these sequences have been annotated and were found to be dispersed within intragenic and intergenic regions of the human genome [1]. Many of the UCRs can be classified as nonexonic, with the others being either exonic or possibly exonic. The extreme conservation of these regions indicate that genetic variations in UCRs are under a negative selection that is much stronger than it is for protein coding genes [2]. Given their extreme conservation for the last 400 million years, it has been postulated that these regions must have biological functions essential to mammalian cells. However, the possible functions of UCRs have remained largely enigmatic since their discovery, with some exceptions. For example, a few ultraconserved regions have been functionally implicated in transcriptional enhancement, alternative splicing, or nonsense mediated decay (RNA surveillance) mechanisms [3-5].

Recently UCRs were identified as the origin of novel transcripts [6]. Many of them appear to be non-coding RNAs. Their roles are largely unknown. However, they may exert their function as non-coding RNAs that regulate other RNAs through RNA: RNA interactions [6] or may have the regulatory roles described for long intergenic non-coding RNAs (lincRNAs), which form complexes with proteins and participate in chromatin regulation [7]. UCRs are frequently located at fragile sites and genomic regions involved in cancer development [6]. Few additional studies have profiled UCR-derived transcripts and they showed that transcripts from these regions are distinctively expressed in human cancer tissues [8]. Similar to known cancer-related genes, some ucRNAs have been found to undergo CpG island hypermethylation-associated silencing [9]. However, only very few of them have been described functionally in cell culture models. For example, uc.73 was found to influence apoptosis in colon cancer cells [6] and uc.338 to inhibit the growth of hepatocellular carcinoma cells [10]. Together these studies indicate a candidate oncogenic function of ucRNAs in the pathogenesis of cancer.

In the present study, we took an exploratory approach that was aimed to establish a gene expression profile for UCR-encoded transcripts in human prostate cancer. To search for the possible functions of ucRNAs as RNAs that target protein-coding genes through inhibitory RNA: RNA interactions, RNA loop-loop interactions were computationally modeled to discover ucRNA:mRNA binding pairs. We also tested whether androgen exposure or epigenetic drug therapy may affect ucRNA transcript expression. Lastly, we correlated ucRNA expression with global mRNA and microRNA (miR) expression to examine relationships between them that may yield new insight into the function of ucRNAs. These studies showed that ucRNAs are aberrantly expressed in prostate cancer and their expression can be responsive to androgen and epigenetic drugs.

## Results

### ucRNA expression profiles in prostate tumors

**Figure 1:**
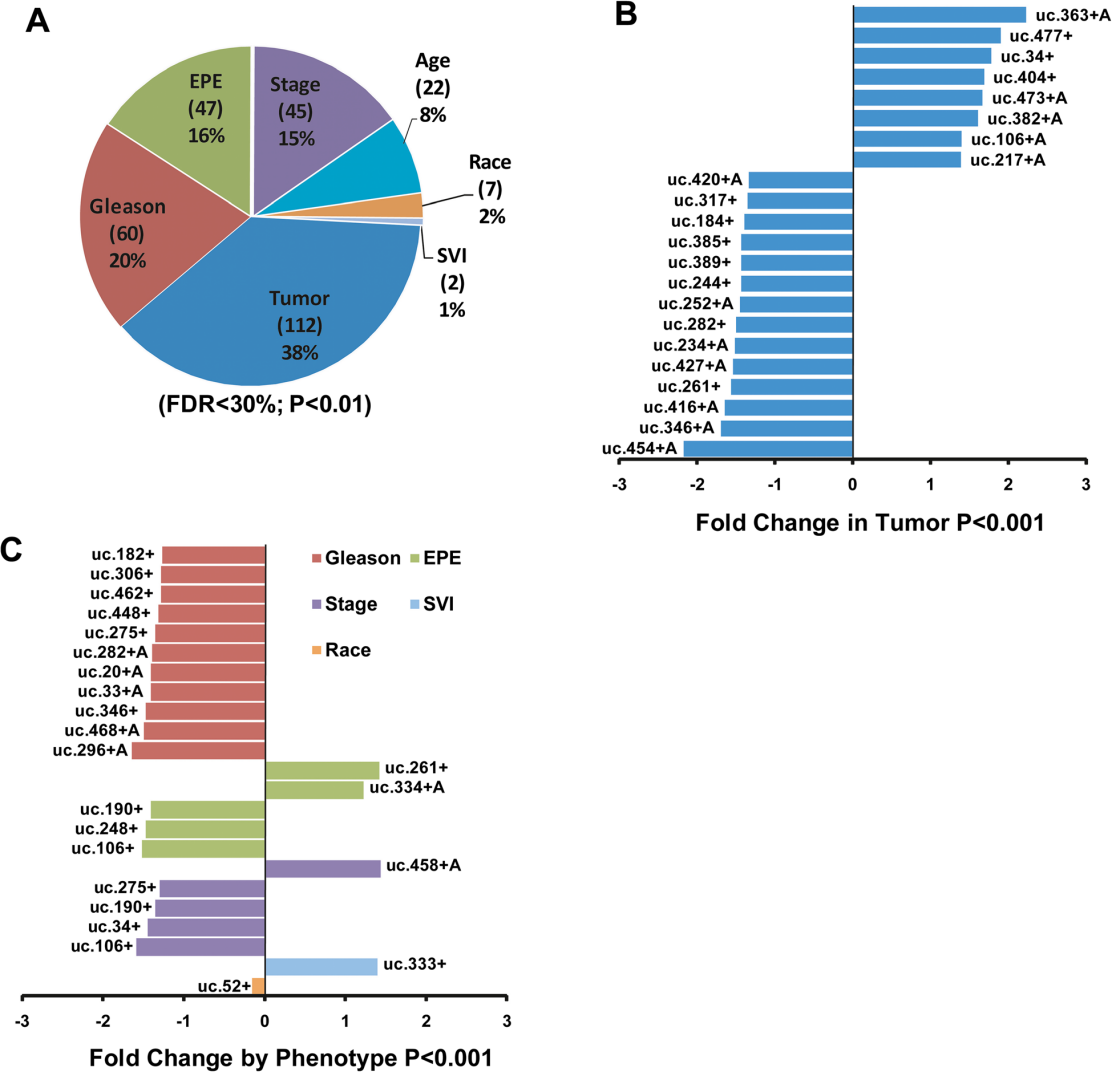
**Global expression patterns of ucRNAs in human prostate cancer. (A)** The summary chart shows percent and number (in parenthesis) of ucRNAs found to be altered in human prostate cancer for the various class comparisons at the indicated cutoff. EPE = extraprostatic extension of the disease and SVI = seminal vesicle invasion. **(B)** Most significantly altered ucRNAs in tumor and **(C)** by clinical characteristics of the prostate cancer patients. The *P* < 0.001 (all FDR < 5%) significance level defines an altered transcript in **(B)** and **(C)**. Class comparisons in **(A)** and **(C)**: Tumor vs. normal; high (≥7) vs. low (≤6) Gleason sum score; pT3 vs. pT2 (stage); EPE yes vs. no; SVI yes vs. no; African-American vs. European-American (race); ≤ age 60 versus ≥ age 61 (age at prostatectomy).

**Figure 2:**
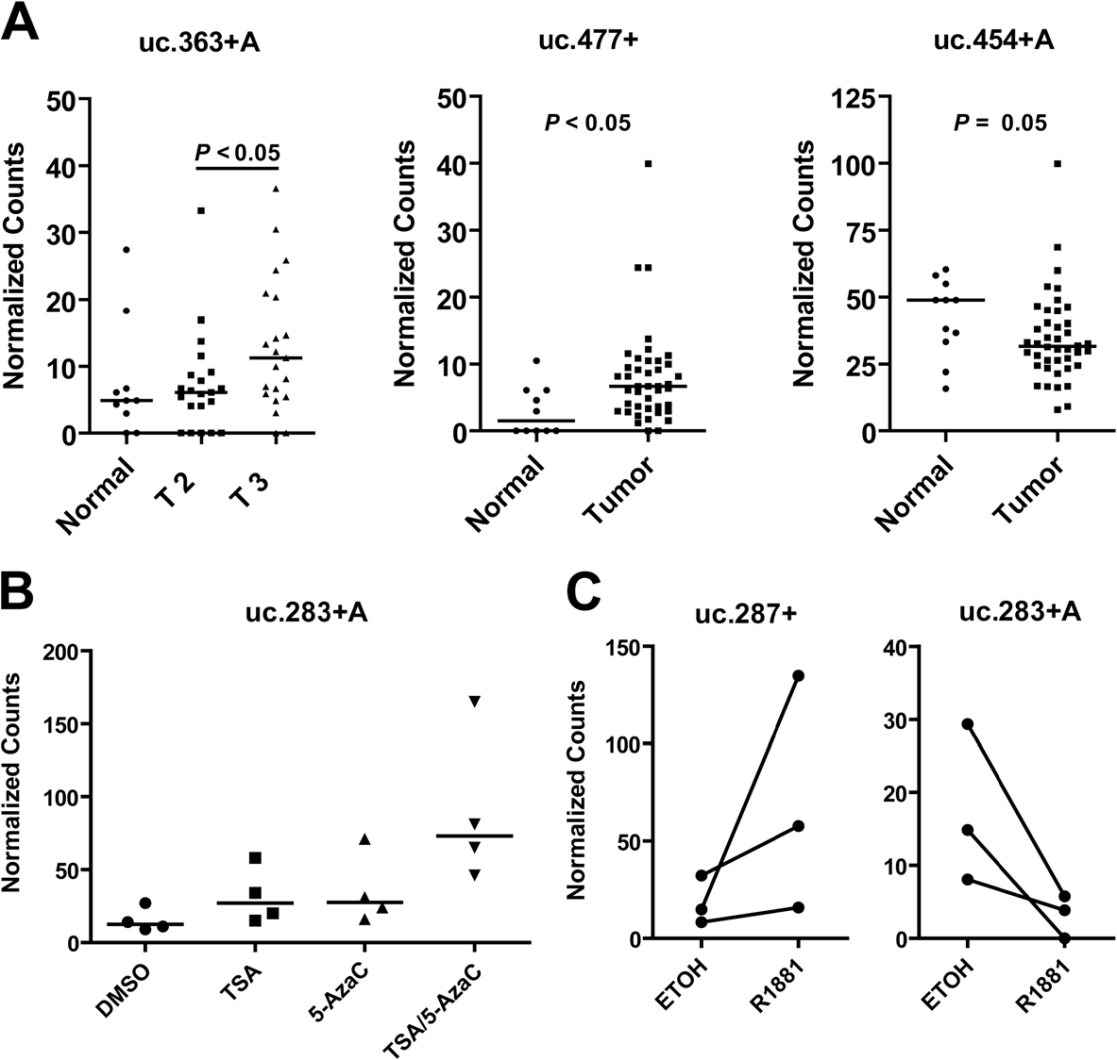
**ucRNA expression in prostate tumors and regulation of ucRNAs by epigenetic drugs and the androgen receptor ligand, R1881, using NanoString nCounter-based quantification. (A)** Up-regulation of uc.363 + A and uc.477 and down-regulation of uc.454 + A in primary prostate tumors versus adjacent non-cancerous tissues. Expression was measured using the NanoString nCounter system, as described in the methods. The uc.363 + A transcript was found to be most notably up-regulated in stage 3 tumors and expression between stage 2 and stage 3 tumors was significantly different (consistent with the array data). **(B)** Nanostring-based analysis of uc.283 + A expression in LNCaP cells treated with epigenetic drugs. Expression of uc.283 + A was significantly up-regulated after treatment with 5-AzaC and TSA *(P* < 0.05 with one-way ANOVA and Tukey’s posthoc test), with a significant trend from untreated to mono treatment to combination treatment (*P* < 0.05). **(C)** Nanostring-based analysis of uc.287+ and uc.283 + A expression in LNCaP cells treated with R1881. Three independent experiments were performed with 10 nM R1881. Shown are the expression changes from untreated to treated for the experiments (mean of n = 3 per individual experiment; *P* < 0.05; paired *t*-test).

The expression of 962 candidate transcripts (ucRNAs) encoded by the 481 known UCRs (sense and antisense transcripts: 962) was evaluated with a custom microarray that was used previously to examine ucRNA expression profiles in human leukemia and in colon and hepatocellular cancer [6,9,10]. We analyzed 57 tumors and 7 non-cancerous prostate tissues. The characteristics of the cancer patients are shown in Table 1. Applying Significance Analysis of Microarrays for class comparison [11], numerous ucRNAs were found to be differentially expressed after controlling for false discovery [false discovery rate (FDR) in tables] between tumor and non-cancerous tissue (see Additional file 1: Table S1 for top-ranked ucRNAs), between tumors with high and low Gleason grade (≥ 7 versus ≤ 6) (Additional file 2: Table S2), and between tumors that showed an extraprostatic extension of the disease (EPE) and those that did not (Additional file 3: Table S3). Many of the transcripts were significantly differently expressed at a stringent FDR < 5%. Similar numbers of transcripts were up- and down-regulated in the tumor versus non-tumor contrast, with the relative change not exceeding the 3-fold range. This and other contrasts are captured in Figure 1A that summarizes the differentially expressed ucRNAs for seven contrasts using a more relaxed FDR < 30%. To further emphasize the most significantly altered ucRNAs, we applied a stringent *P* < 0.001 (FDR < 5%) cutoff to all class comparisons (Figures 1B and 1C), showing that both sense and antisense transcripts (antisense: +A) were detected among these differently expressed transcripts. Among these, only few ucRNAs were differently expressed in more than one contrast. For example, uc.106+ was up-regulated in tumors (1.8-fold vs. non-tumor; *P* = 0.005), but tended to be down-regulated by 30% to 40% in tumors with high Gleason grade (vs. low grade; *P* = 0.009) and EPE (vs. without EPE; *P* < 0.001). At a *P* ≤ 0.01 cutoff for differently expressed genes, uc.34+ and uc.346+ were both down-regulated in high Gleason grade disease and also high stage disease. Because a high Gleason score frequently coincides with high stage disease and presence of EPE, the data suggest that these ucRNAs are generally down-regulated with disease progression. The expression of selected ucRNAs was confirmed with NanoString technology, as shown in Figure 2A for three of the most differently expressed UCR-encoded transcripts. The Nanostring nCounter gene expression system captures and counts individual RNA transcripts and has been described in details [12]. Exploratory Prediction Analysis of Microarrays identified a 60 ucRNA probeset signature (Additional file 4: Figure S1) that could robustly separate non-tumor (class error rate = 0) from tumor tissue (class error rate = 5%) with 3 out of 57 tumors being misclassified, further highlighting the differences between cancerous and non-cancerous tissue.

**Table 1:** Clinical characteristics of the prostate cancer patients in the study

	**All patients**
	**(n = 57)**
Age at prostatectomy (years) [median (range)]	61 (46 – 73)
Largest individual tumor nodule (grams) [median (range)]	1.6 (0.2 – 3.0)
Pathological stage	**N (%)**
2	28 (49)
3 – 4	29 (51)
Gleason grade sum score	
< 7	13 (23)
≥ 7	44 (77)
Extraprostatic disease extension	
No	33 (58)
Yes	24 (42)
Seminal vesicle invasion	
No	47 (82)
Yes	10 (18)
Race/ethnicity of the patients*	
African-American	29 (51)
European-American	28 (49)

* By self-report.

### Correlation between ucRNA and host gene expression

**Figure 3:**
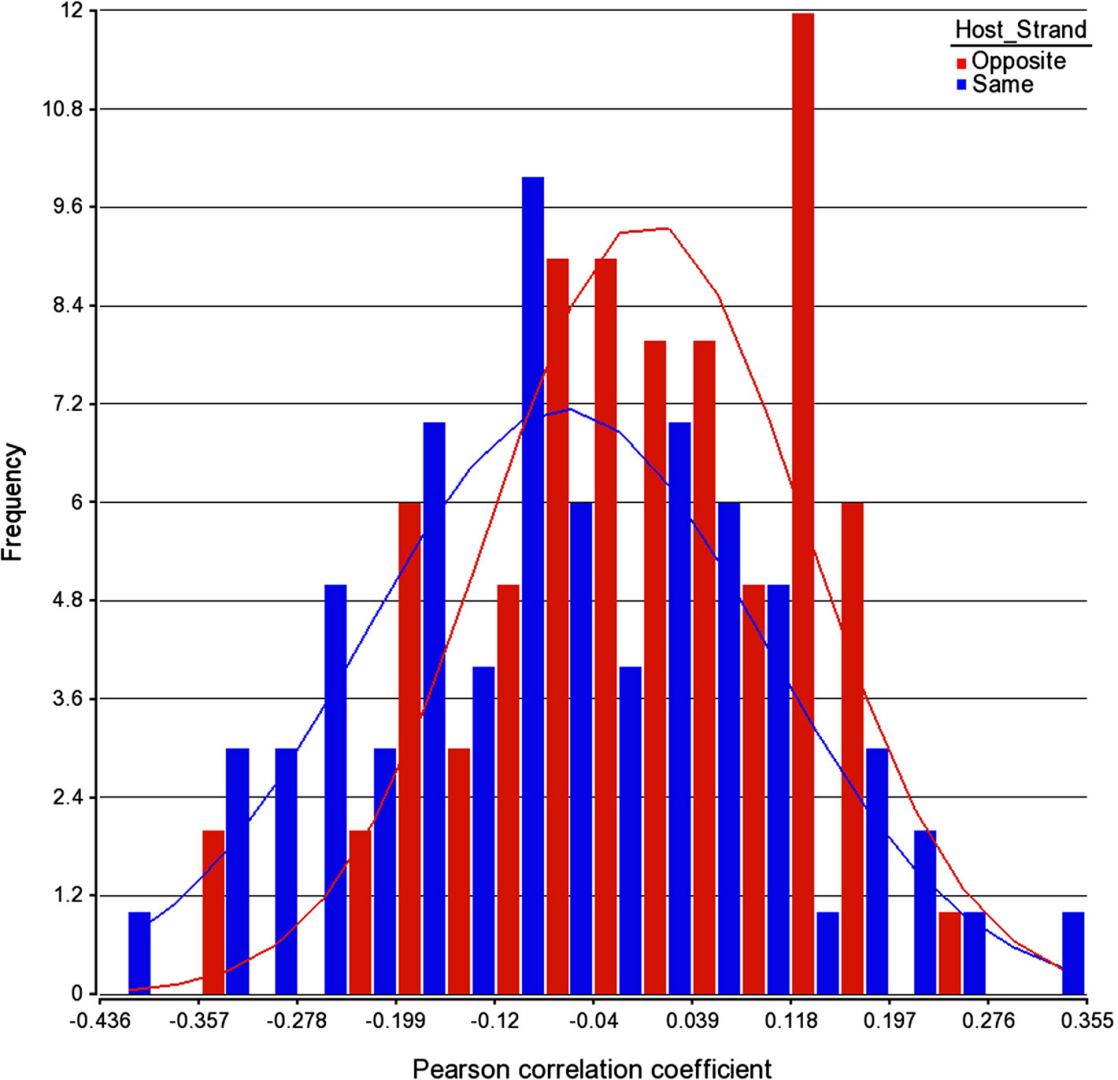
**Lack of association between ucRNA expression and the expression of the respective “host” mRNAs in prostate tumors.** Shown is the distribution curve of the Pearson correlation coefficients between tumor-altered ucRNAs and the corresponding UCR-encoded mRNAs. The correlation coefficient for the ucRNA-mRNA pairs (n = 146) are clustered within the −0.2 to 0.2 range, independent whether pairs are sense (blue) or antisense (red) transcribed. UCR-encoded mRNAs are defined as mRNA transcripts with a coding region that at least partially overlaps with the UCR locus. Tumor-altered ucRNAs were defined as being differently expressed between tumor and adjacent non-cancerous tissue at the *P* ≤ 0.01 significance level.

Because a subset of the 481 UCRs overlaps with the coding region for mRNAs, we wanted to characterize the relationship between the expression of ucRNAs and UCR-encoded mRNAs. Using available Affymetrix GeneChip mRNA expression data for all tumor tissues, we performed a correlation analysis between the expression of those ucRNAs, whose expression was altered in prostate cancer, and corresponding UCR-encoded mRNAs. This analysis revealed that the expression of the existing ucRNA-mRNA pairs (n = 146) did not correlate significantly with one another, as shown in Figure 3, by the normal (or Gaussian) distribution with a bell-shaped probability density function centered around a correlation coefficient of 0. Taken together, our findings indicate that ucRNA expression is principally independent from the expression of UCR-encoded mRNAs in the human prostate. This is in contrast to the significant positive correlation seen between miRs and miR host genes, where miRs tend to be transcribed in parallel with their host mRNA transcripts [13].

### Transcription of UCRs is influenced by epigenetic mechanisms

To identify epigenetically regulated ucRNAs, we profiled LNCaP cells that were treated with the DNA hypomethylating agent, 5-azacytidine (5-AzaC), the histone deacetylase inhibitor, trichostatin A (TSA), or the combination of the two. 5-AzaC can induce the expression of genes that are repressed by DNA hypermethylation [14,15]. Histone deactylase inhibition alters the acetylation status in a number of substrates, including histones and transcription factors, and has been shown to affect the expression of mRNAs and miRs [16]. LNCaP cells were treated with 5-AzaC and/or TSA for 36 hrs. Total RNA was collected from these cells and processed as described under methods and changes in global ucRNA expression were examined by microarray analysis. Using the OSU-CCC 4.0 array, we identified six ucRNAs that were consistently up-regulated in the 3 treatment groups (5-AzaC only, TSA only, and the combination of both), indicating that these UCRs are epigenetically silenced in prostate cancer (Table 2). Among them was uc.283 + A, an antisense transcript encoded by the intergenic UCR283, which was previously found to be silenced by promoter CpG hypermethylation in human colon cancer cells [9]. We confirmed with NanoString technology that uc.283 + A was up-regulated in 5-AzaC- and TSA-treated LNCaP cells. This analysis indicated the most significant up-regulation of uc.283 + A in cells treated with combined 5-AzaC and TSA (Figure 2B).

**Table 2:** ucRNA expression changes in LNCaP cells in response to treatment with epigenetic drugs

	Fold Change^*,†^					
**ucRNA**	**5-AzaC**	**TSA**	**5-AzaC + TSA**	**Type‡, host gene, host strand**	**Overlap with mRNA**	**Antisense to mRNA**
uc.308 + A	3.02	4.66	4.03	n, BTRC, sense	Yes	Yes
uc.434 + A	2.25	3.84	3.59	n, SKOR2, sense	Yes	
uc.241 + A	2.03	2.31	3.12	n, no gene	No	
uc.283 + A	1.90	2.17	2.39	n, no gene	No	
uc.285+	1.82	2.14	2.37	e, CCAR1, sense	Yes	
uc.85+	1.63	2.03	1.96	n, no gene	No	

* Fold change: reference are the untreated cells (solvent only); †Cutoff for inclusion: *P* value (*t*-test) < 0.01 treatment versus control; ‡ Type is based on UCSC human genome data, UCSC version hg19, NCBI build 37 coordinates, where, n = nonexonic and e = exonic.

### Androgen-responsive UCRs

To assess putative androgen-responsive UCRs, we surveyed the global expression of ucRNAs in androgen-responsive LNCaP prostate cancer cells after 24 hr stimulation with the androgen receptor ligand and agonist, R1881. We preformed the same experiment in androgen-insensitive DU145 cells to have a negative control for our system. This experiment identified a number of ucRNAs that were R1881-responsive in the LNCaP cells (Table 3). Treatment of the androgen-insensitive DU145 cells with R1881 did not yield any significant changes in ucRNA expression, indicating that the effect of R1881 in LNCaPs is androgen receptor pathway-specific. The group of altered ucRNAs contained both up- and down-regulated transcripts (e.g., uc.287+ and uc.283 + A), consistent with mRNA profiling data showing that R1881 induces and represses protein-coding RNAs in a 24 hr stimulation period [17]. The intergenic uc.287+ was found to be the most consistently up-regulated transcript, which was also confirmed in an additional experiment with NanoString-based expression analysis (Figure 2C). We further examined the androgen-responsive ucRNA loci for the presence of candidate androgen receptor binding sites. This analysis found that several loci, including the UCR encoding uc.287+, contain putative binding sites within 1000 base pairs up- or down-stream of the UCR (Additional file 5: Table S4), indicating that uc.287+ is a candidate direct target of the androgen receptor signaling pathway.

**Table 3:** ucRNA expression changes in LNCaP cells after treatment with R1881

**ucRNA**	Fold Change*	**FDR (%)**	***P *value**^**†**^	**Type‡, host gene, host strand**	**Overlap with mRNA**	**Antisense to mRNA**
uc.287+	1.83	7.8	0.034	p, no gene	Yes	
uc.445 + A	1.44	28.8	0.042	n, no gene	No	
uc.134+	1.39	28.8	0.029	n, RSRC1	Yes	
uc.240+	1.37	28.8	0.019	n, no gene	Yes	Yes
uc.249 + A	0.80	13.7	0.026	n, no gene	No	
uc.349+	0.78	13.1	0.021	n, DACH1, antisense	Yes	Yes
uc.204+	0.76	9.7	0.025	n, no gene	No	
uc.135+	0.65	1.6	0.01	e, MECOM, antisense	Yes	Yes
uc.31+	0.61	1.6	0.025	p, no gene	Yes	
uc.410 + A	0.58	0.0	0.022	n, no gene	No	
uc.344+	0.57	0.0	0.037	e, HOXC5, sense	Yes	Yes
uc.283 + A	0.52	0.0	0.032	n, no gene	No	
uc.283+	0.26	0.0	0.008	n, no gene	No	

* Fold change: reference are the untreated cells (solvent only); †Cutoff for inclusion: *P* value (*t*-test) < 0.05 for all ucRNAs; ‡ Type is based on UCSC human genome data, UCSC version hg19, NCBI build 37 coordinates, where n = nonexonic, e = exonic, and p = possibly exonic.

### Prediction of RNA loop-loop interactions to identify mRNAs and pathways that are candidate targets of ucRNAs

A previous report indicated that UCR-encoded transcripts may exert their function as non-coding RNAs that regulate other RNAs through RNA: RNA interactions [6]. However, little is known about these interactions although their description can provide clues for mechanisms and functional analysis of otherwise uncharacterized ucRNAs with an altered expression in diseases like prostate cancer. To explore this possible function of UCR-encoded transcripts, we modeled direct ucRNA:mRNA interactions based on sequence complementarity and predicted RNA loop-loop interactions as dynamic functional motifs. This exploratory approach identified almost 1400 possible ucRNA:mRNA binding pairs representing 302 different sense and antisense ucRNAs and 1058 different mRNAs (Additional file 6: Table S5), using a folding energy threshold cutoff at −10 kcal/mol. To infer possible functions of ucRNAs via interactions with mRNAs, we queried all mRNAs predicted to form loop-loop interactions with ucRNAs for an enrichment pattern in Gene Ontology (GO)-defined biological processes and KEGG-defined pathways. This analysis revealed a ucRNA target enrichment among mRNAs for GO processes related to ion binding (*P* = 9.4 × 10^-7^, GO:0043167) and KEGG pathways related to calcium signaling (*P* = 8.5 × 10^-3^). We also queried specific ucRNAs and found, for example, that uc.454 + A, which was the most down-regulated ucRNA in prostate tumors, was predicted to directly interact with Ras signaling pathway-related transcripts, like RIN2 and RAB37.

### Correlation between tissue ucRNAs and expression of mRNAs and miRs

Because ucRNAs may influence the expression level and function of other RNAs, we examined available tumor mRNA and miR expression data (~ 13,000 mRNAs, 238 mature and 143 precursor miRs) to assess whether ucRNA expression is associated with particular cancer-related processes in these tumors as indicated by the mRNA expression profiles, or with the expression of cancer-related miRs. The correlation analysis was performed for selected ucRNAs (uc.106+, uc106 + A, uc.248+, uc.454 + A, uc.346+, uc.363 + A, uc.477+, and uc.477 + A). This analysis revealed a prominent inverse correlation between uc.106 + A expression and the expression of many interferon pathway genes such as IRF7, ISG15, ISG20, OAS1-3, and PTPN22 in prostate tumors (Additional file 7: Table S6). On the other hand, uc.346 transcript levels correlated with global miR expression more so than transcript levels of the other ucRNAs and showed a distinct inverse association with several miRs including miR-143 (−.77 to -.85 across probesets; *P* < 10^-10^), miR-27 (−.74 to -.75; *P* < 10^-9^), miR-21 (−.69 to -.75; *P* < 10^-8^), and miR-16 (−.68 to -.74; *P* < 10^-7^) expression levels and a positive association with miR-373 (.84 to .88; *P* < 10^-13^) and miR-9 (.78 to .85; *P* < 10^-10^) in the prostate tumors. Our analyses also suggested that sense and antisense transcribed ucRNAs act independently. Accordingly, we did not find that mRNA and miR expression profiles, which were significantly correlated with sense strand UCR expression, were also correlated with antisense strand expression from this location, neither negatively or positively.

### A gene expression signature induced by knockdown of uc.106+ in LNCaP cells

**Figure 4:**
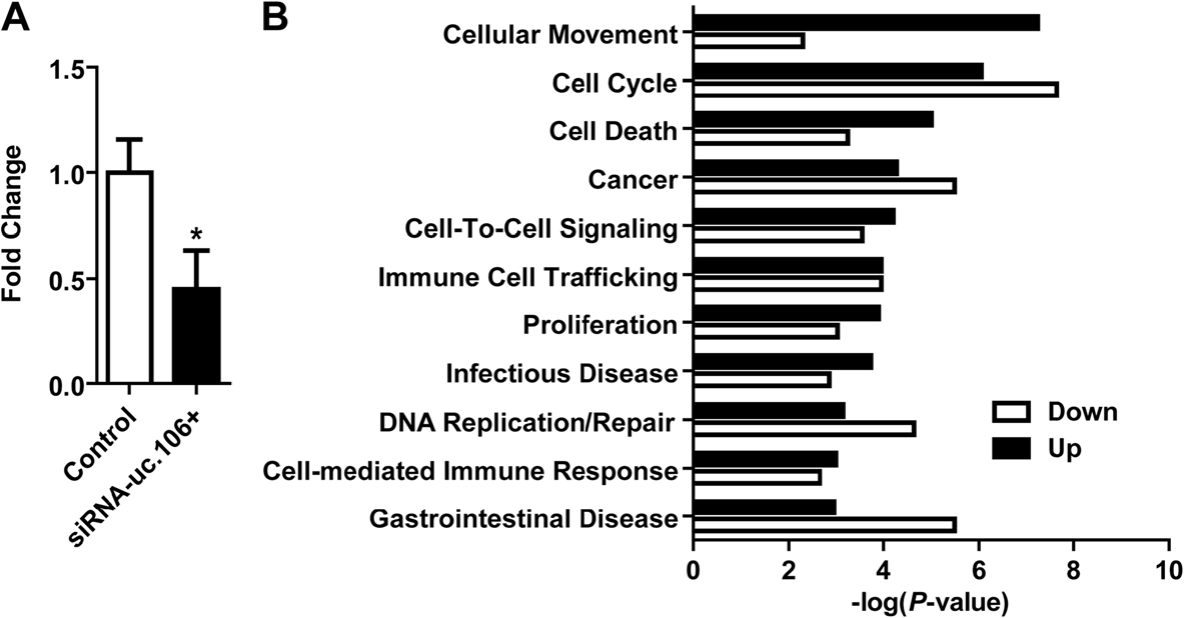
**Knockdown of uc.106+ alters expression of cell proliferation, motility, and inflammatory genes in LNCaP cells.(A)** Knockdown of uc.106+ in LNCaP cells at 24 hr after transfection with siRNA targeting uc.106+ (or scrambled siRNA as control). * *P* < 0.05 (versus control). **(B)** Pathway analysis with the 200 top-ranked genes which were significantly up- or down-regulated after siRNA transfection across 3 independent experiments. The analysis shows significant clustering of these genes in several biological processes including cell proliferation and movement, and inflammation.

The ucRNA expression profiles of prostate tumors indicated that UCR106 may encode for cancer-related transcripts. To gain insight into the possible functional role of RNAs derived from UCR106, LNCaP cells were transfected with a siRNA designed to target the sense UCR106 transcript, uc.106+, which was up-regulated with tumor development but was down-regulated in cancer progression (high Gleason score tumors and tumors with EPE). Knockdown of endogenous uc.106+ was confirmed by strand specific qRT-PCR (Figure 4A). To evaluate whether uc.106+ knockdown may affect cell function, global expression analysis was then carried out 24 hr post transfection of LNCaP cells using Affymetrix GeneChips. Numerous genes were found to be up- and down-regulated in the siRNA-transfected cells when compared with cells transfected with scrambled siRNA (control). These genes clustered in distinct pathways related to proliferation and cell movement, and also cancer and immune response (Figure 4B). While preliminary, the experiment indicates that UCR106-encoded transcripts may affect cellular transcription pattern in prostate cancer cells, consistent with the proposed interaction between ucRNAs and other RNAs.

## Discussion

Key functions of non-coding RNAs in human cancer have recently been described and several classes of non-coding RNAs (e.g., miRs, ucRNAs, lincRNAs, snoRNAs) are now known whose expression is dysregulated in the disease because of existing oncogenic stimuli, genome amplifications and deletions, mutations, and epigenetic silencing [18,19]. Here, we examined the expression of transcripts encoded by 481 UCRs in prostate cancer, defined as ucRNAs, and found that ucRNAs can be detected in the cancerous human prostate and show a disease-specific expression pattern. When we compared the expression of ucRNAs in prostate cancer with other human cancer types, e.g., leukemia, colon cancer, liver cancer, and neuroblastoma [6,10,20,21], we did not find a common ucRNA expression signature among them. Thus, ucRNA expression is rather tissue-specific and cancer type-specific, which is reminiscent of miR expression patterns in solid human tumors [22,23]. However, it appears that even fewer ucRNAs than miRs are commonly dysregulated across tumor types, as we could not identify any commonly dysregulated ucRNAs between our study and other ucRNA studies. We also observed that only very few UCRs encoded for transcripts that were associated with both disease onset and progression. Among them were UCR106 and UCR346 that were found to encode for sense and antisense transcripts (uc.106+, uc.106 + A, uc.346+, uc.346 + A) and whose expression was dysregulated in primary tumors and with disease stage and Gleason grade. Previous studies identified robust miR signatures only for the tumor versus non-cancerous tissue contrast, but did not find those signatures when studying tumor grade and disease stage differences in prostate cancer [24-26]. Our study of ucRNAs concurs with these findings.

The underlying mechanisms that cause cancer-specific ucRNA expression are largely unknown but may include mutational events and epigenetic regulation. Some UCRs are regulated by epigenetic silencing, as was shown recently [9], which is consistent with the well established regulation of other non-coding RNAs, like miRs, by this mechanism [27]. Our study identified 6 ucRNAs whose expression significantly increased after treatment of LNCaP cells with the two epigenetic drugs, 5-AzaC and TSA. Of those, uc.283 + A was up-regulated by these drugs in both our study and the study by Lujambio and coworkers, who also showed that the uc.283 + A locus is silenced by CpG hypermethylation in a human colon cancer cell line [9]. We did not examine the uc.283 + A locus in more detail but the consistent finding in the two studies (present study and [9]) identifies uc.283 + A as an epigenetically regulated transcript. UCR283 is located in an intergenic genomic region and does not overlap with the coding region for any mRNA. While epigenetic regulation of UCR283 could be observed in the LNCaP cells, we did not find that transcription from UCR283 was significantly altered in human prostate tumors but noticed that two of the six epigenetically regulated ucRNAs, uc.241 + A and uc.285+, were down-regulated in these tumors when compared with adjacent non-cancerous tissue. Thus, future investigations are needed to define the importance of ucRNA silencing in human prostate cancer biology.

Another important mechanism of gene regulation in prostate biology and prostate cancer progression is the activation of the androgen receptor signaling pathway [28]. It has been shown that non-coding RNAs like miRs are regulated by androgen signaling [25,29-31]. We examined the effect of the androgen receptor agonist, R1881, in androgen-sensitive LNCaP cells and surveyed global expression changes by ucRNAs. Our experiment was restricted to one dose of R1881 and future experiments would have to investigate R1881 effects on UCR transcription using a broader dose range and a larger panel of cell lines. Nevertheless, this exploratory analysis identified several ucRNAs that were induced by R1881, though only the up-regulation of uc.287+ was robust. In contrast, multiple ucRNAs were found to be significantly down-regulated in response to R1881. Among them was uc.283+, which was the most significantly repressed transcript. We did not find that any of these androgen-responsive ucRNAs are differently expressed in primary human prostate tumors, when compared with non-cancerous prostate tissue, or were associated with Gleason grade or disease stage, nor did we find that these ucRNAs were, with the exception of uc.135+, described in other ucRNA studies [6]. Thus, they do not appear to be cancer-associated ucRNAs in the primary disease. Nevertheless, it remains a possibility that these transcripts are differently expressed between castration-resistant and castration-sensitive prostate tumors, which we could not investigate.

Like with most non-coding RNAs other than miRs, our current knowledge with respect to the function of ucRNAs is very limited. Only a few full length ucRNA transcripts have been described. Because of these limitations, we used siRNA to knockdown uc.106+ expression and also decided to apply computational analyses to predict RNA-RNA interactions between ucRNAs and mRNAs based on the available coding sequence from the 481 described UCR loci, and to link these interactions to possible functions of the ucRNAs. In addition, we applied correlation analyses of ucRNA, mRNA, and miR expression data to discover functional associations between selected ucRNAs and both miRs and mRNAs. These are exploratory tools that can yield new insight into ucRNAs in the absence of other supporting information in this largely unexplored research field. Down-regulation of uc.106+ generated a robust gene expression profile in LNCaP cells, indicating that uc.106+ is a functional transcript and suggesting that UCR106 encoded transcripts may have a function in prostate cancer. Future research is needed to clone candidate non-coding transcripts encoded by UCR106 to allow functional analysis of them in phenotypic assays. Other analyses also revealed a significant enrichment for predicted ucRNA:mRNA interactions in processes related to ion binding and calcium signaling, suggesting that ucRNAs may target calcium signaling processes. Our approach also showed that expression of uc.106 + A (antisense transcript of UCR106) in prostate tumors may influence the interferon signaling pathway by either a direct or indirect mechanism in prostate tumors, as was indicated from the inverse relationship between uc.106 + A expression and expression of multiple interferon pathway genes. Notable, UCR106 also encodes a DNA damage-regulated gene and ATPase, termed *OLA1* or Obg-like ATPase, which is a putative GTP-binding protein involved in mitochondrial function and regulation of the oxidative stress response [32,33]. UCR106 is located in an intronic region of this gene and we did not find a correlation between *OLA1* expression and the expression of either uc.106+ or uc.106 + A. While these are only few examples of candidate functions for ucRNAs in prostate cancer, our exploratory work shows that these approaches can be useful in potentially uncovering the biology of ucRNAs in cancer biology.

## Conclusions

This first study of ucRNA expression in the cancerous human prostate shows a disease-specific expression pattern for this class of transcripts. Future studies are needed to further define the functional implication of aberrantly expressed ucRNAs in human prostate cancer pathogenesis.

## Material and methods

### Clinical samples

Fifty-seven fresh-frozen primary prostate tumors, 7 non-cancerous prostate tissues from prostatectomy (not paired to the tumors in the study), and additional patient information were received from the National Cancer Institute (NCI) Cooperative Prostate Cancer Tissue Resource (CPCTR). All tissues were collected between 2002 and 2004 through CPCTR. Non-tumor tissues were collected from patients whose prostate was resected because of a prostate cancer diagnosis. These tissues did not contain tumor per assessment by a pathologist. Tumors were surgically resected adenocarcinomas from patients who had not received any therapy prior to prostatectomy. Their characteristics are described in Table 1. These tumors were macrodissected by a CPCTR pathologist who also confirmed that the frozen tissue specimen is tumor. Tissue collection and its use was reviewed and approved by the institutional review boards of the participating institutions in the CPCTR and the NIH Office of Human Subjects Research. Written informed consent was obtained from all donors.

### RNA isolation

Total RNA was isolated using TRIZOL reagent according to the manufacturer’s instructions (Invitrogen, Carlsbad, CA). RNA integrity for each sample was confirmed with the Agilent 2100 Bioanalyzer (Agilent Technologies, Palo Alto, CA).

### Expression microarrays

The Ohio State University Comprehensive Cancer Center (OSU-CCC) Version 2.0 custom microarray was used for ucRNA and miR expression profiling and the Affymetrix GeneChip HG-U133A 2.0 microarray for mRNA expression profiling, following previously published protocols [6,34,35]. The OSU-CCC microarray was developed with a total of 962 probesets representing sense and antisense sequences for the 481 human UCR as in http://www.soe.ucsc.edu/∼jill/ultra.html. An updated UCR annotation (location, type, transcripts) based on UCSC human genome data, UCSC version hg19, NCBI build 37 coordinates, can be found for selected ucRNAs in Additional file 8: Table S7. For each UCR two 40-mer probes were designed, one corresponding to the sense genomic sequence (named “+”) and the other to the complementary sequence (named “+A”). Each oligo was printed in duplicate in two different slide locations, and therefore quadruplicate numerical values were available for analysis. Several thousand (3484) blank spots were used for background subtraction. Labeling and hybridization of ucRNA transcripts were performed as described previously [34]. Briefly, 5 μg of RNA from each tissue sample was labeled with biotin by reverse transcription using random octomers. Hybridization was carried out onto the OSU-CCC Version 2.0 microarray, which contained the 962 UCR probes, 238 probes for mature miRs, and 143 probes for precursor miRs. More information about this custom microarray can be found under the ArrayExpress accession number: A-MEXP-258. Hybridization signals were detected by biotin binding of a Streptavidin-Alexa647 conjugate (one-color signal) using a GenePix 4000B scanner (Axon Instruments). Images were quantified using the GenePix Pro 6.0 (Axon Instruments). Labeling and hybridization of mRNAs from the same tissues were performed according to Affymetrix standard protocols (Santa Clara, CA). Briefly, 5 μg of total RNA was reverse transcribed with an oligo (dT) primer that has a T7 RNA polymerase promoter at the 5^′^ end. Second-strand synthesis was followed by cRNA production with incorporation of biotinylated ribonucleotides using the BioArray High Yield RNA Transcript Labeling Kit T3 from Enzo Life Sciences (Farmingdale, NY). The labeled cRNA was fragmented and hybridized to Affymetrix GeneChip HG-U133A 2.0 arrays. This array contains 22,283 probe sets that represent approximately 13,000 human protein-coding genes. Hybridization signals were visualized with phycoerythrin-conjugated streptavidin (Invitrogen) and scanned using a GeneChip Scanner 3000 7 G (Affymetrix). In accordance with Minimum Information About a Microarray Experiment (MIAME) guidelines, we deposited the CEL files for the microarray data and additional patient information into the GEO repository (http://www.ncbi.nlm.nih.gov/geo/). Normalized and raw data files for the ucRNA, miR, and mRNA profiling data have been uploaded to GEO (http://www.ncbi.nlm.nih.gov/geo) under GEO accession GSE7055, as described previously [35]. The OSU-CCC 4.0 microRNA array was used for profiling of ucRNAs in prostate cancer cells treated with R1881 or epigenetic drugs. Normalized and raw expression data from these cell-based experiments were deposited into GEO under the accession number GSE31620. GEO also describes the OSU-CCC 4.0 platform under the accession number GPL14184.

### Data normalization and statistical analysis of microarray data

Median-centric normalization was used for the OSU-CCC 2.0 custom microarray. Affymetrix arrays were normalized using the robust multichip analysis (RMA) procedure [36]. To generate lists of differently expressed transcripts (ucRNAs, miRs, mRNAs) between classes (e.g. tumors versus non-cancerous tissue), the resulting dataset was subjected to the Significance Analysis of Microarrays [11]. A description of SAM can be found at http://www-stat.stanford.edu/~tibs/SAM. We generated gene lists based on both *P* values from two-sided t-tests and intended false discovery rates (FDRs) and report both for our findings of differently expressed genes as fold change compared to the reference with *P* value and FDR. The FDR calculation followed the method described by Storey and Tibshirani [37]. False discovery control by calculating FDRs is a http://statistical method used in http://multiple hypotheses testing to correct for http://multiple comparisons, especially relevant for high-throughput data such as expression microarrays where changes in many genes are measured at the same time. Although the range of a FDR acceptable for a particular study depends on the actual dataset, in general, a FDR (or q-value) < 5% for a differently expressed transcript is usually used as cutoff for a statistical significant finding but FDRs at 5-10% or even higher can be meaningful. This has been described in more details in the references we cited and the URL for the SAM method. We used Prediction Analysis of Microarrays [38] to classify tissues into tumor and non-tumor tissue based on their ucRNA expression pattern. Pathway analysis was performed with Ingenuity (Ingenuity® Systems, http://www.ingenuity.com). For the analysis of the cell-based experiments with OSU-CCC 4.0 arrays, the raw GPR data files were imported into BRB array tools. Median normalization was carried out and spots were filtered for low intensities and minimum fold-change. Probe sets with 50% missing or filtered data were excluded. Class comparisons between treatments were carried out using the randomized block univariate *t*-test across all samples.

### NanoString nCounter evaluation of ucRNA expression

The nCounter gene expression system utilizes a novel digital technology that is based on a direct measurement of gene expression (http://www.nanostring.com). The technology uses molecular barcodes and single molecule imaging to detect and count transcripts. It has been described in details [12]. Nanostring nCounter codesets for ucRNAs were designed and produced based on the published sequences for the corresponding UCRs and are listed in Additional file 9: Table S8. Expression analysis was performed according to the written protocol provided by NanoString Technologies (Seattle, WA). Briefly, a 5 μl aliquot containing 100 ng of denatured total RNA was added to 20 μl of master mix consisting of equal amounts of Reporter CodeSet solution and hybridization buffer. Five μl of Capture ProbeSet were added to the mix the hybridization was performed overnight at 65°C. Post-hybridization processing was performed at the NCI/CCR DNA Sequencing and Digital Gene Expression Core Facility. Raw counts were normalized to internal mRNA levels of the *GUSB* gene.

### Treatment of prostate cancer cells with R1881

For sex hormone depletion, DU145 and LNCaP human prostate cancer cells were placed in phenol red-free RPMI 1640 with 5% charcoal-treated FBS for 48 hours. Cells were then treated with either 10 nM R1881 (methyltrienolone; PerkinElmer Life Sciences, Waltham, MA) or solvent (ethanol). After 24 hrs, cells were harvested and total RNA was isolated. This experiment was repeated five times and the RNA was processed for microarray-based expression analysis as described under **“**Expression microarrays”. The same treatment protocol was used to validate uc.287+ up-regulation with the Nanostring nCounter gene expression system. A search for putative androgen receptor binding sites in or nearby selected UCR locations was performed with the Genomatix software (München, Germany).

### Treatment of prostate cancer cells with 5-Aza 2^′^deoxycytidine (5-AzaC) and Trichostatin A (TSA)

LNCaP cells were treated with 5-azacytidine (5-AzaC) and/or trichostatin A (TSA) (Sigma, Saint Louis, MO, USA) following similar schemes reported by others [39]. Briefly, cells were plated at 1x10^6^ cells per 10 cm^2^ for 48 hrs and then treated with dimethylsulfoxide (DMSO) as a control and 5-AzaC (5 mM), and/or TSA (0.3 mM). For combined treatments, TSA was added after 12 hrs of pre-treatment with 5-AzaC or control. After 36 hrs, cells were harvested for RNA extraction. N = 4 for 5-AzaC + TSA, n = 5 for all others.

### Knockdown of uc.106+ with siRNA and gene expression profiling

LNCaP cells were transiently transfected with siRNA oligos (Qiagen, Valencia, CA) targeting the uc.106+ transcript target sequence, ATGGTGTGAAGTATAGGTTAA, encoded by UCR106 using lipofectamine 2000, as described by the manufacturer (Invitrogen), or with scrambled siRNA oligos as negative control (non-targeting control). Strand-specific quantification of uc.106+ knockdown: Single strand cDNA synthesis was performed using the ThermoScript RT-PCR system (Invitrogen) following the manufacturer’s suggested procedures. Briefly, 1 μg of DNase I (Amp Grade 1 U/μl; Invitrogen) treated RNA, 10 μM of sense uc.106 region specific primers (PrimerDesign Ltd., Southampton, UK) along with U6 TaqMan® MicroRNA Assay primers (Applied Biosystems), and 10 mM dNTP were denatured by incubating at 65°C for 5 minutes. Next, a mixture of 5x cDNA synthesis buffer, 0.1 M DTT, and RNAseOUT was added and the reaction was incubated at 60 min at 50°C. The reaction was then terminated by heating to 85°C for 5 minutes and then incubated with RNase H for 37°C for 20 minutes. Real-time PCR was performed using standard protocols on an Applied Biosystem’s 7900HT Sequence Detection System (SDS) using 2x SYBR green PCR master mixes (Applied Biosystems) for uc.106+ or Taqman 2x Universal PCR Master Mix for quantification of the internal control U6. Cycle number threshold (C_T_) was calculated automatically within SDS software and fold change of gene expression was calculated using the comparative C_T_ method. For genome-wide mRNA expression analysis following uc.106+ knockdown, total RNA was isolated with the the TRIzol reagent at 24 hr post siRNA transfection. For arrays, 250 ng of total RNA was amplified using the Affymetrix GeneChip 3’ IVT Express Kit. After fragmentation, aRNA was hybridized onto Affymetrix GeneChip HG-U133A 2.0 according to Affymetrix standard protocols.

### Prediction of loop-loop RNA interactions

Putative ucRNA-mRNA interactions were evaluated based on predicted loop-loop RNA interactions using the RNAfold program from the Vienna package for the folding analysis [40] and derived custom scripts for the analysis of loop-loop interactions, similar to previously described general procedures to predict these interactions [41]. The RNAfold software was used to predict secondary structures for transcribed UCR sequences and for mRNAs in the REFseq collection. Loop subsequences of these structures were then predicted and possible pairs of ucRNA-mRNA sequences were evaluated for their joint structure and folding energy. For predicted interactions, the folding energy threshold was set at greater than −10 kcal/mol and the number of bonds in the ucRNA-mRNA pair had to be more than 75% within the evaluated sequence.

### Statistical analysis beyond microarrays

We used t-tests (paired and unpaired), ANOVA, and the Pearson correlation test in our statistical analysis. All statistical tests were two-sided, and an association was considered statistically significant with *P* values < 0.05.

## Abbreviations

UCR: Ultraconserved regions; miR: microRNA; ucRNA: UCR-encoded transcript; EPE: Extraprostatic disease extension; FDR: False discovery rate.

## Competing interests

The authors have declared that no conflict of interest exists.

## Authors’ contributions

Designed study: SA, GAC, CMC. Performed experiments: RSH, RLP, CGL. Performed analyses: MY, NV, RSH, SV, RMS, AJS, KV. Provided tools: DE, CGL. Wrote manuscript: RSH, SA. All authors read and approved the final manuscript.

## Supplementary Material

Additional file 1: Table S1 Listing the top-ranked ucRNAs differently expressed between cancerous and non-cancerous human prostate tissue. Click here for file

Additional file 2: Table S2 Listing the top-ranked ucRNAs associated with Gleason score. Click here for file

Additional file 3: Table S3 Listing the ucRNAs associated with extraprostatic disease extension. Click here for file

Additional file 4: Figure S1 Summarizing the shrunken centroid differences (*dik*) for each of a 60 ucRNA probeset classifier that differentiated cancer from non-cancerous tissue in the human prostate. Click here for file

Additional file 5: Table S4 Listing putative androgen-related receptor binding sites in R1881-responsive ultraconserved regions. Click here for file

Additional file 6: Table S5 Listing predicted interactions between ucRNA transcripts (sense and antisense transcribed) and mRNAs. Click here for file

Additional file 7: Table S6 Summarizing the correlation of ucRNA106 + A with mRNA expression in human prostate tumors. Click here for file

Additional file 8: Table S7 That contains the updated annotation of selected UCR-encoded transcripts based on UCSC version hg19, NCBI build 37 coordinates. Click here for file

Additional file 9: Table S8 Containing the target sequences in selected ucRNAs for the Nanostring probe design. Click here for file
