# Supplementary material for: Transcription signatures encoded by ultraconserved genomic regions in human prostate cancer
Source: Mol Cancer. 2013 Feb 14;12:13. doi: 10.1186/1476-4598-12-13 (PMC3626580; doi:10.1186/1476-4598-12-13)
Supplement: Additional file 1: Table S1 — Listing the top-ranked ucRNAs differently expressed between cancerous and non-cancerous human prostate tissue. [file 1476-4598-12-13-S1.pdf]

Supplementary Table 1. Top-ranked ucRNAs differentially expressed between tumor (n = 57) and nontumor tissue (n = 7)

ucRNA	Fold Change*	FDR (%)	*P* value	Type‡, host, host strand	Overlap mRNA	Antisense to mRNA
uc.363+A	2.22	0	6.76E-04	n, EST, antisense	No	
uc.477+	1.9	0	6.83E-05	e, PLP1 exonic, sense/RAB9B intronic, antisens	Yes	
uc.374+A	1.81	0	2.64E-03	p, MIPOL1 intronic, sense	Yes	Yes
uc.106+	1.81	1	5.20E-03	p, OLA1 (PTD004) intronic, antisense	Yes	Yes
uc.34+	1.78	0	4.24E-04	e, PTBP2 exonic/intronic, sense	Yes	
uc.22+	1.74	0	2.45E-03	n, FAF1 intronic, antisense	Yes	Yes
uc.158+	1.71	1	4.60E-03	n, no gene	No	
uc.404+	1.69	0	9.47E-04	n, no gene	No	
uc.473+A	1.67	0	7.48E-06	e, NLGN3 introinc/exonic, sense	Yes	Yes
uc.362+A	1.67	0	1.02E-03	p, EST, sense	No	
uc.483+A	1.63	0	2.05E-03	p, TBC1D5 intronic, antisense	Yes	
uc.382+A	1.61	0	8.04E-05	p, no gene	No	
uc.89+	1.56	0	1.52E-03	e, PSMD14 intronic/exonic, sense	Yes	
uc.20+A	1.56	1	1.76E-03	p, EST intronic, sense/antisense	No	
uc.215+	1.52	1	2.05E-03	n, GLI3 intronic, antisense	Yes	Yes
uc.153+A	1.5	0	3.38E-03	e, TNPO1 intronic/exonic, sense	Yes	Yes
uc.248+	1.48	2	1.98E-03	n, no gene	No	
uc.308+A	1.47	4	3.17E-03	p, BTRC intronic, sense	Yes	Yes
uc.63+	1.43	0	1.19E-03	e, XPO1 intronic, antisense	Yes	Yes
uc.356+A	1.41	5	2.13E-03	e, MBNL2 intronic/exonic, sense	Yes	Yes
uc.106+A	1.4	0	6.44E-04	p, OLA1 (PTD004) intronic, antisense	Yes	
uc.217+A	1.39	4	3.08E-04	p, VSTM2A intronic/exonic, antisense	Yes	
uc.3+	1.37	6	1.17E-03	p, FLJ20321 intronic, antisense	No	Yes
uc.47+	1.37	1	3.42E-03	n, no gene	No	
uc.4+	1.35	5	4.64E-03	n, EST intronic, antisense	No	
uc.33+	1.3	3	4.52E-03	e, PTBP2, sense	Yes	
uc.20+	1.28	4	4.96E-03	p, EST intronic, sense/antisense	Yes	
uc.354+A	1.26	4	2.68E-03	p, EST intronic, sense	No	
uc.349+	0.81	7	1.60E-03	n, DACH intronic, antisense	Yes	Yes
uc.327+	0.8	10	3.46E-03	n, ELP4 intronic, sense	Yes	
uc.181+A	0.77	2	1.46E-03	n, RANBP17 intronic, sense	Yes	Yes
uc.158+A	0.77	2	1.95E-03	n, EST, antisense	No	
uc.420+A	0.75	2	9.37E-04	e, DDX5 intronic/exonic, antisense	Yes	
uc.317+	0.74	3	1.75E-04	n, MGMT intronic, sense	Yes	
uc.390+	0.73	1	2.40E-03	e, MAP2K5 intronic/exonic, sense	Yes	
uc.184+	0.72	1	1.86E-04	e, CPEB4, sense	Yes	
uc.244+	0.7	1	8.23E-06	n, no gene	No	
uc.389+	0.7	0	6.24E-05	n, FLJ12476 intronic, sense	Yes	
uc.385+	0.7	1	5.02E-04	p, MEIS2 intronic, antisense	No	Yes
uc.252+A	0.69	1	2.91E-04	n, BNCS intronic, antisense	Yes	
uc.282+	0.67	0	3.17E-04	e, GRIN1 exonic/intronic, sense	Yes	
uc.249+	0.67	0	1.65E-03	n, no gene	No	
uc.1+	0.67	0	3.94E-03	p, PEX14 intronic, sense	Yes	
uc.234+A	0.66	0	2.74E-05	p, EST, sense	No	

uc.427+A	0.65	0	3.72E-06	p, ESTs, antisense/sense	No	
uc.261+	0.64	0	8.65E-09	n, no gene	No	
uc.249+	0.64	0	4.09E-03	n, no gene	No	
uc.291+	0.63	1	1.93E-03	p, C10orf11, sense	Yes	
uc.416+A	0.61	0	6.42E-06	e, HOXB5 intronic/exonic, antisense	Yes	
uc.346+A	0.59	0	6.71E-04	p, EST intronic, sense	No	
uc.241+A	0.57	0	4.80E-03	n, no gene	No	
uc.345+A	0.56	0	1.21E-03	e, HOXC4 intronic/exonic, sense	Yes	Yes
uc.118+A	0.54	0	1.36E-03	n, no gene	No	
uc.73+	0.5	0	3.87E-03	p, BC017741 intronic, antisense	Yes	Yes
uc.366+	0.48	0	4.58E-03	e, STRN3 intronic/exonic, antisense	Yes	Yes
uc.454+A	0.46	0	2.97E-05	e, SLC23A1 exonic, antisense	Yes	
uc.359+A	0.3	0	1.87E-03	e, NOVA1 exonic, antisense	Yes	

* Fold change, reference is nontumor tissue

‡ Type is based on the original annotation of UCRs (http://users.soe.ucsc.edu/~jill/ultra.html) with n = nonexonic, e = exonic, and p = possibly exonic.
