# Supplementary material for: Transcription signatures encoded by ultraconserved genomic regions in human prostate cancer
Source: Mol Cancer. 2013 Feb 14;12:13. doi: 10.1186/1476-4598-12-13 (PMC3626580; doi:10.1186/1476-4598-12-13)
Supplement: Additional file 2: Table S2 — Listing the top-ranked ucRNAs associated with Gleason score. [file 1476-4598-12-13-S2.pdf]

Supplementary Table 2. Top-ranked ucRNAs differentially expressed between high (≥7) and low (≤6) Gleason sum score

ucRNA	Fold Change*	FDR (%)	*P* value	Type‡, host, host strand	Overlap with mRNA	Antisense to mRNA
uc.454+A	1.5	4.8	1.56E-03	e, SLC23A1 intronic/exonic, antisense	Yes	
uc.315+A	1.27	8.4	2.83E-03	n, no gene	No	
uc.21+A	1.26	6	1.04E-03	n, FLJ14442 intronic, antisense	Yes	
uc.262+A	1.2	8.4	4.96E-03	n, no gene	No	
uc.465+	0.8	9.1	2.75E-03	p, POLA intronic, sense	Yes	
uc.263+	0.8	3.5	1.52E-03	e, HNRPK exonic/intronic, antisense	Yes	
uc.182+	0.79	2.3	4.40E-04	n, RANBP17 intronic, sense	Yes	
uc.282+A	0.78	5.3	1.17E-03	e, GRIN1 exonic/intronic, sense	Yes	Yes
uc.306+	0.78	2.3	2.75E-04	p, BTRC intronic, sense	Yes	
uc.462+	0.78	3.5	1.35E-04	p, POLA intronic, sense	Yes	
uc.215+	0.76	3.7	3.09E-03	n, GLI3 intronic, antisense	Yes	Yes
uc.172+A	0.76	4.8	2.88E-03	n, AK130941 intronic, antisense	Yes	
uc.448+	0.76	2.3	8.07E-04	n, EST intronic, sense	Yes	
uc.275+	0.74	0	3.07E-04	n, PBX3 intronic, sense	Yes	
uc.43+A	0.73	3.1	3.07E-03	n, AKT3 intronic, antisense	Yes	
uc.282+A	0.72	1.6	2.12E-04	e, GRIN1 exonic/ intronic, sense	Yes	Yes
uc.483+A	0.72	2.3	1.51E-03	p, TBC1D5 intronic, antisense	Yes	
uc.20+A	0.71	1.6	6.06E-04	p, EST intronic, sense/antisense	Yes	
uc.33+A	0.71	0	1.81E-04	e, PTBP2, sense	Yes	Yes
uc.339+	0.71	2	1.05E-03	p, no gene	No	
uc.348+	0.7	2.3	1.45E-03	n, DACH intronic, antisense	Yes	Yes
uc.117+A	0.69	1.6	1.52E-03	n, no gene	No	
uc.346+	0.68	0	6.92E-06	p, EST, sense	No	
uc.204+	0.68	1.6	4.31E-03	n, no gene	No	
uc.468+A	0.67	0	4.70E-04	p, no gene	No	
uc.145+A	0.67	1.6	3.65E-03	n, no gene	No	
uc.167+	0.64	0	3.40E-03	n, EST intronic, antisense	No	
uc.269+A	0.64	0	1.52E-03	n, KIAA1608, antisense	Yes	
uc.296+A	0.61	0	3.41E-04	n, no gene	No	

* Fold change, reference is low Gleason sum score.

‡ Type is based on the original annotation of UCRs (http://users.soe.ucsc.edu/~jill/ultra.html) with n = nonexonic, e = exonic, and p = possibly exonic.
