# Supplementary material for: Transcription signatures encoded by ultraconserved genomic regions in human prostate cancer
Source: Mol Cancer. 2013 Feb 14;12:13. doi: 10.1186/1476-4598-12-13 (PMC3626580; doi:10.1186/1476-4598-12-13)
Supplement: Additional file 4: Figure S1 — Summarizing the shrunken centroid differences (dik) for each of a 60 ucRNA probeset classifier that differentiated cancer from non-cancerous tissue in the human prostate. [file 1476-4598-12-13-S4.pdf]

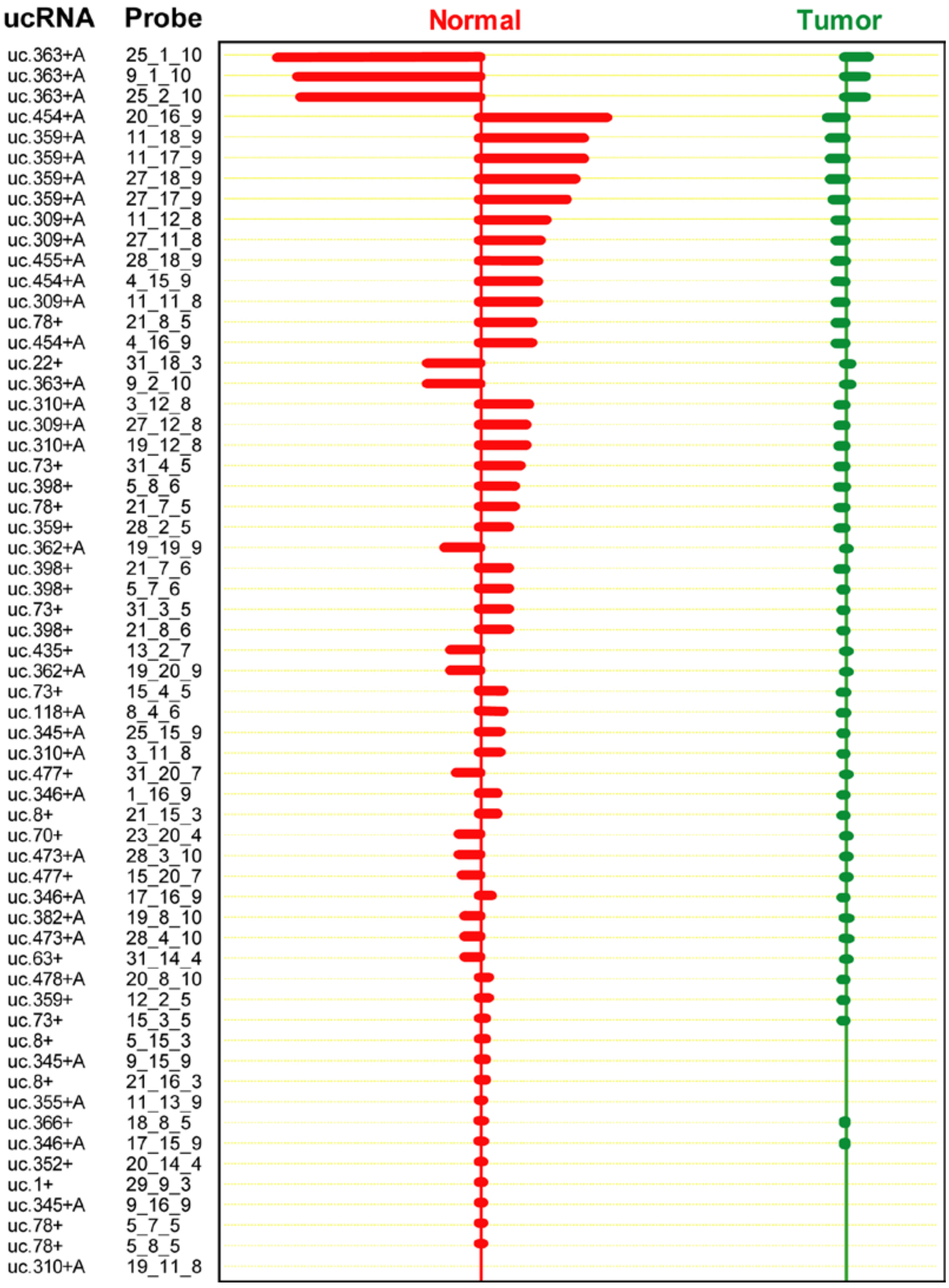

Supplementary Figure 1. Diagnostic 60 ucRNA probeset classifier. Prediction Analysis of Microarray (PAM) was used to identify ucRNAs that differentiate between tumor and non-tumor tissues. Shown are the shrunken centroid differences (d_{ik}) for each of the 60 ucRNA probes.
