# Supplementary material for: Transcription signatures encoded by ultraconserved genomic regions in human prostate cancer
Source: Mol Cancer. 2013 Feb 14;12:13. doi: 10.1186/1476-4598-12-13 (PMC3626580; doi:10.1186/1476-4598-12-13)
Supplement: Additional file 5: Table S4 — Listing putative androgen-related receptor binding sites in R1881-responsive ultraconserved regions. [file 1476-4598-12-13-S5.pdf]

Supplementary Table 4. Putative androgen-related receptor binding sites in R1881-reponsive UC regions

UC region	Binding Site	Start position	End position	Matrix Similarity	Sequence	Strand
uc.134	GRE.01	675	693	0.856	gagttattatatGTGCtta	+
uc.135	ARE.02	807	825	0.923	ctgccacttggtGTACtga	+
uc.135	GRE.02	807	825	0.851	tcaGTACaccaagtggcag	-
uc.135	GRE.02	2032	2050	0.842	aatGCACattgtgtattct	+
uc.204	GRE.03	1047	1065	0.893	ttcctcctgtaTGTTctga	-
uc.204	GRE.03	1460	1478	0.893	gcggctcacgtTGTTcaga	-
uc.204	ARE.03	1666	1684	0.892	tgtccacgggctGTCCtcc	+
uc.204	PRE.01	989	1007	0.885	tttggctttgtTGTTcttg	+
uc.204	GRE.02	1666	1684	0.849	ggaGGACagcccgtggaca	-
uc.204	GRE.02	1786	1804	0.839	gcgGGACaggcagtggcca	-
uc.240	ARE.03	578	596	0.891	gctgatccagatGTCCtta	+
uc.240	PRE.01	100	118	0.89	aggtgcccaacTGTTctga	+
uc.240	PRE.01	1226	1244	0.887	agagccgaggaTGTTcttt	-
uc.240	PRE.01	564	582	0.882	tcagcctcggtTGTTcttg	-
uc.240	ARE.03	747	765	0.872	ttaggaaattgtGTACttc	+
uc.240	ARE.03	1101	1119	0.871	ctgtctcagcttGTGCtaa	-
uc.240	GRE.02	493	511	0.863	ctgGTACacgcggtcatct	-
uc.240	GRE.02	455	473	0.848	tggGTACacatggtattta	-
uc.240	GRE.02	578	596	0.835	taaGGACatctggatcagc	-
uc.240	GRE.02	747	765	0.826	gaaGTACacaatttcctaa	-
uc.249	ARE.02	1382	1400	0.895	tgatttccttctGTTCcct	+
uc.249	GRE.03	2219	2237	0.889	acacctcactcTGTTccag	-
uc.249	PRE.01	86	104	0.879	catatatgtagTGTTctca	+
uc.249	ARE.03	2657	2675	0.871	ctcgcataaaatGTGCtta	+
uc.249	PRE.01	2298	2316	0.869	tgagaggctcaTGTTctgt	+
uc.283	GRE.01	1102	1120	0.869	aagtttgattttGTTCtca	+
**uc.287**	**ARE.02**	**1259**	**1277**	**0.922**	**gtcttgcctgctGTTCtca**	-
**uc.287**	**GRE.03**	**1164**	**1182**	**0.914**	**aaagtgccaaaTGTTctgc**	-
**uc.287**	**GRE.03**	**1464**	**1482**	**0.892**	**tccttccagatTGTTccca**	-
**uc.287**	**GRE.03**	**152**	**170**	**0.886**	**tttgtcctccgTGTTcact**	+
**uc.287**	**GRE.01**	**143**	**161**	**0.862**	**gcagtaatctttGTCCtcc**	+
**uc.287**	**ARE.01**	**2079**	**2097**	**0.834**	**ctagaaccccaTGTTgtct**	+
**uc.287**	**GRE.02**	**1164**	**1182**	**0.822**	**gcaGAACatttggcacttt**	+
uc.31	ARE.02	2214	2232	0.931	tttttgcctcctGTTCttt	-
uc.31	GRE.03	265	283	0.899	tatgctcggcaTGTTccag	+
uc.344	GRE.01	2240	2258	0.853	aacgtcacttctGTCCtgg	+
uc.349	PRE.01	503	521	0.852	ttatgttttaaTGTTctat	-
uc.410	GRE.01	2240	2258	0.853	aacgtcacttctGTCCtgg	+
uc.445	ARE.03	279	297	0.899	cttgttcccactGTGCtga	-
uc.445	ARE.03	2842	2860	0.893	gttgcacaggctGTGCact	+
uc.445	GRE.02	2842	2860	0.89	agtGCACagcctgtgcaac	-
uc.445	GRE.01	146	164	0.85	tgtgtggggtgtGTCCtgt	-
uc.445	GRE.02	279	297	0.821	tcaGCACagtgggaacaag	+
uc.445	GRE.02	2514	2532	0.821	gttGCACagtgggtctgtg	+

Note: Search included UC regions and corresponding 1kb flanking sequences of ucRNAs found to be altered in R1881-treated cells, using Genomatix software (München, Germany). Genomatix matrix family (V$GREF) included the androgen receptor binding sites and related glucocorticoid responsive elements: ARE.01: RGNACRnngTGTTCT; ARE.02: DNWCWtnnTGTYCT; PRE.01: GNACNnknTGTTCT; GRE.01:
