# Supplementary material for: Transcription signatures encoded by ultraconserved genomic regions in human prostate cancer
Source: Mol Cancer. 2013 Feb 14;12:13. doi: 10.1186/1476-4598-12-13 (PMC3626580; doi:10.1186/1476-4598-12-13)
Supplement: Additional file 9: Table S8 — Containing the target sequences in selected ucRNAs for the Nanostring probe design. [file 1476-4598-12-13-S9.pdf]

Supplementary Table 8: Nanostring probe design.

Probe Name	Target Sequence
uc.106+	GCTATTAAAGCTGAATGGTGATGGTGTGAAGTATAGGTTAAATTGGGTGAAATTAAAGCAAATTACTCCGGGATGTGGAAATCTGAAAATAGAAACCACC
uc.283+	TGAAATTTTCAAGTCAGGGGCGCGGGATTGGATCAAATCACATAAACTGCAAAAAAAGCAAGTCTAATGGCGCATAATTGGTTCTGTAATAGCATTACAC
uc.283+A	CCCCTTTAATATCATAAAGGCTTTCCGCCAGGGAAGCACTTAATAGTGCAGGGGGCCGTAACGGGCTATTATCCTGGTGTAATGCTATTACAGAACCAAT
uc.287+	TTGCAGATAATTTTCAGTGCTCAGATCTCTGTGTAGTATTGATGGGGAGGGTGGTTGATTAGGACCTTGGGAATTGTGCTTGATGGGAACAATCTGGAAG
uc.363+A	CCCTATGTGGCTTGAATGACAGTTGTTAAGTTGAAAAAGGGACAGTGTGATGGGAATGCCACTTTAAATCTCAATTACCTCCTCCACTCCAATCAGCTTG
uc.454+A	TCTATGGAAAAGACTGTTAAGCTGTCAAATAGTGAACTACCTCGGTAGTTCCCGATGCCGTGATCATGGTTTTGGTCTCGTTTCTTTTAGGTTACCCCTG
uc.477+	ATTCTTTGGAGCGGGTGTGTCATTGTTTGGGAAAATGGCTAGGACATCCCGACAAGGTGATCATCCTCAGGATTTTGTGGCAATAACAAGGGGTGGGGGA
GUSB	CGGTCGTGATGTGGTCTGTGGCCAACGAGCCTGCGTCCCACCTAGAATCTGCTGGCTACTACTTGAAGATGGTGATCGCTCACACCAAATCCTTGGACCC
